# Development and validation of a prognostic nomogram for malignant esophageal fistula based on radiomics and clinical factors

**DOI:** 10.1111/1759-7714.14115

**Published:** 2021-10-14

**Authors:** Chao Zhu, Jialin Ding, Songping Wang, Qingtao Qiu, Youxin Ji, Linlin Wang

**Affiliations:** ^1^ Department of Oncology Qingdao Central Hospital Qingdao China; ^2^ Department of Radiation Oncology Shandong Cancer Hospital and Institute Jinan China; ^3^ Department of Radiation Physics Shandong Cancer Hospital and Institute Jinan China

**Keywords:** esophageal cancer, esophageal fistula, prognostic factors, radiomics

## Abstract

**Background:**

The current study aimed to comprehensively analyze the clinical prognostic factors of malignant esophageal fistula (MEF). Furthermore, this study sought to establish and validate prognostic nomograms incorporating radiomics and clinical factors to predict overall survival and median survival after fistula for patients with MEF.

**Methods:**

The records of 76 patients with MEF were retrospectively analyzed. A stepwise Cox proportional hazards regression model was employed to screen independent prognostic factors and develop clinical nomograms. Radiomic features were extracted from prefistula CT images and post fistula CT images. Least absolute shrinkage and selection operator (LASSO) regression and Cox regression algorithm was used to filter radiomic features and avoid overfitting. Radiomic signature was a linear combination of optimal features and corresponding coefficients. The joint prognostic nomograms was constructed by radiomic signatures and clinical features. All models were validated by Harrell's concordance index (C‐index), caliberation and bootstrap validation.

**Results:**

For overall survival, age, prealbumin, KPS and interval between diagnosis of esophageal cancer and fistula were identified as independent prognostic factors and incorporated into the clinical nomogram. Age, prealbumin, serum albumin, KPS and neutrophil proportion were selected for the clinical nomogram of post fistula survival. The C‐index of overall survival nomogram was 0.719 (95% CI: 0.645–0.793) and that was 0.722 (95% CI: 0.653–0.791) in the post fistula survival nomogram. The radiomic signature developed by radiomic features of prefistula CT showed a significant correlation with both overall survival and post fistula survival. The C‐index of joint nomogarm for overall survival and post fistula survival was 0.831 (95% CI: 0.757–0.905) and 0.77 (95% CI: 0.686–0.854), respectively. The calibration curve showed the joint nomograms outperformed the clinical ones.

**Conclusions:**

The study presents nomograms incorporating independent clinical risk factors and radiomic signature to predict the prognosis of MEF. This prognostic classification system has the potential to guide therapeutic decisions for patients with malignant esophageal fistulas.

## Graphical abstract

The study presents nomograms incorporating independent clinical risk factors and radiomic signature to predict the prognosis of malignant esophageal fistula. This risk classification system has the potential to guide therapeutic decisions for patients with malignant esophageal fistula.

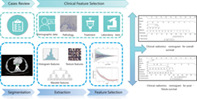

## INTRODUCTION

Malignant esophageal fistula, with a reported incidence of 4.8%–22% in patients without surgical intervention, is a serious complication of advanced esophageal cancer (EC).^1^, ^2^, ^3^ Depending on the site of fistula, it can be divided into esophageal respiratory fistula (ERF), esophageal mediastinal fistula (EMF) and esophageal aortic fistula (EAF),which occurs acutely and most patients are undiagnosed before death.^4^ EMF and ERF can cause severe mediastinal infection or pneumonia leading to ARDS, sepsis, septic shock, and even death. Previous studies have indicated that the prognosis of malignant esophageal fistula caused by EC is extremely poor, with a median survival time of 1–6 weeks after fistula diagnosis.^5^, ^6^ However, because of the low incidence, there is a lack of studies on MEF prognosis. Recently, a retrospective study reported the prognostic factors of esophageal fistula, but some important potential prognostic factors were not analyzed, such as Karnofsky performance score (KPS), radiotherapy and interval between diagnosis of EC and fistula.^7^ Another important reason for the lack of research is the low discrimination of clinical factors in the prognosis evaluation.

Radiomics, a new image processing technology, converts medical images into high‐dimensional data, namely radiomic features.^8^ These features provide information about tumor phenotype and microenvironment, which are relatively independent and interrelate with traditional clinical factors. They can complement each other and provide more information about the heterogeneity of tumors.^8^

The aim of this study was to comprehensively analyze the clinical prognostic factors of esophageal fistula and to construct a prognostic predictive nomogram incorporating radiomics and clinical factors.

## METHODS

### Patients

This retrospective study of consecutive patients with biopsy‐proven EC was approved by the ethics committee of Shandong Cancer Hospital and Institute (Approval no. 2021003193) and the requirement for informed consent was waived. All esophageal fistula patients were identified from the medical records database of Shandong Cancer Hospital and Institute between October 2018 to September 2020. Esophageal fistula was diagnosed by endoscopy or meglumine diatrizoate esophagography, but not by computed tomography (CT) alone.

Exclusion criteria were esophageal surgery, other malignant tumors, esophageal fistula induced by medical injury, lack of contrast‐enhanced CT at initial diagnosis of EC or one month after fistula diagnosi, poor CT image quality or serious artifacts.

All CT images derived from archiving and communication system (PACS) were processed in the format of DICOM (Digital Imaging and Communications in Medicine). Equipment parameters: Philips CT scanner (Brilliance iCT 128, Philips Medical System), tube voltage 120 kV, tube current 368 mAs, slice thickness 5 mm, pixel spacing (0.78125, 0.78125), and image matrix 512 × 512.

### Follow‐up and definition of variables

Follow‐up information and survival data were collected from the most recent medical records and telephone enquiries. The endpoint of this study was overall survival (OS1) and survival time after fistula diagosis (OS2). OS1 was defined as the period from the date of admission to the death date regardless of specific causes of death. OS2 was defined as the period between diagnosis of esophageal fistula and death.

All laboratory parameters: peripheral blood leukocyte count, peripheral blood lymphocyte count, peripheral blood neutrophil count, neutrophil proportion, serum albumin, serum prealbumin, were collected within one week after the diagnosis of esophageal fistula. KPS and body mass index (BMI) were within one week before or after the diagnosis of esophageal fistula. The eighth edition of the American Joint Committee on Cancer (AJCC) staging manual was used for the staging of all patients.

### Development and validation of clinical nomograms

Cox proportional hazards univariate regression model was used to screen the prognostic factors. Variables with *p* < 0.15 were included in multivariate regression analysis. A nomogram model was developed with independent prognostic factors. Performance of the nomogram was assessed by Harrell's concordance index (C‐index), calibration curve and bootstrapping validation.

For a more parsimonious prediction model, we approximated the full model by using a stepwise regression algorithm. The calculated risk scores was the estimated linear predictive value of the approximate full model, and the input mode of all candidate variables was exactly the same as that of the full Cox model.^9^ X‐tile software (Version: 3.6.1, URL: https://x-tile.software.informer.com/download/) was used to identify the best cutoff value of risk scores to classify patients into three risk groups. Survival curves were depicted by Kaplan–Meier method, and survival differences were compared by the log rank test.

### Image segmentation and radiomic feature extraction

Tumor segmentation was performed by 3D slicer, a free open‐source software (Version: 4.10.2, URL: https://www.slicer.org/). Arterial phase CT images were analyzed for tumor segmentation as the arterial phase is more suitable for visualization of esophageal cancer.^10^ The 3D‐labeling region of interest (ROIs) covered the whole tumor, which were manually delineated by an experienced radiologist and confirmed by another radiologist, who were both blinded to the clinical data of all patients. Conditions of delineation were window width 500 and window level 40. The delineation scope included the area of esophageal wall thickening ≥5 mm, excluding intraluminal gas, oral contrast agents and other adjacent organs.

The extraction of all features was implemented by PyRadionomy (URL: https://pyradiomics.readthedocs.io/en/latest/), which is an open source Python package for extracting radiomic features from medical images.

### Radiomic features selection and radiomic signature development

The least absolute shrinkage and selection operator (Lasso) and Cox model was used to avoid overfitting and select the optimal radiomic features from pre‐fistula CT and post fistula images, respectively. Their C‐index was then compared, and the optimized feature set was filtered for the subsequent model construction. Pearson's correlation test was used to exclude collinearity. Radiomic signature was developed as radiomics scores (Radscore) calculated by a linear combination of the selected features that were weighted by their respective coefficients.^11^ To verify the association of radscores with patient survival time, patients were classified into low risk group, middle risk group and high‐risk group according to the radscore threshold, which was identified by X‐tile. The Kaplan Meier method was used to plot survival curves, and log rank test was used to compare survival differences.

### Development and validation of joint nomograms

Joint nomograms were established by radiomic signatures and independent clinical prognostic factors. The method of validation and survival analysis was the same as that of clinical models.

The same methods were applied for the nomograms with the endpoint of OS2.

### Statistical analysis

Statistical analyses was conducted by R software (Version 3.3.3, URL: https://www.r-project.org/). All statistical tests were two‐sided, with a significance level at 0.05. The details of the packages used are described in Appendix Table S1. Restricted cubic spline (RCS) was performed for all clinical continuous variables, and nonlinear variables were converted into categorical variables for statistical analysis.

## RESULTS

### Patient characteristics

The records of 1653 patients with esophageal cancer were reviewed. Ninety‐two patients with esophageal fistula met the inclusion criteria. After excluding patients according to the criteria, a total of 76 patients were admitted to the study. A flowchart is shown in Figure S1.

The histological type of all patients was squamous cell carcinoma. There were 31 cases of EMF, 41 of esophagotracheal fistula, three of esophagopulmonary fistula, and one of esophageal tracheal mediastinal fistula. The overall survival time (OS1) was 11 (IQR, 6,16) months, and the survival time after fistula (OS2) was 113 (IQR, 45,281) days. After a median follow‐up of 20 months, 57 deaths occurred. The KPS of all patients ranged from 20 to 90, with a median of 80. A typical choking cough was seen in 34 of 42 patients with esophagotracheal fistula, one of three patients with esophagopulmonary fistula, and 14 of 31 patients with EMF. The characteristics of enrolled patients are shown in Table 1.

**TABLE 1:** Characteristics of patients included in the analysis

Categories	Characteristics		Patients (*n* = 76)
Tumor	Age	Mean ± SD	60.87 ± 8.84
		Median (IQR, 25th, 75th)	61 (54, 67)
	Length (cm)	Mean ± SD	6.81 ± 2.61
		Median (IQR, 25th, 75th)	6 (5, 8)
	Gender	Female	8 (10.5%)
		Male	68 (89.5%)
	Stage_T	T2	2 (2.6%)
		T3	32 (42.1%)
		T4	42 (55.3%)
	Stage_N	N0	12 (15.8%)
		N1	30 (39.5%)
		N2	27 (35.5%)
		N3	7 (9.2%)
	Stage_M	M1	29 (38.2%)
		M0	47 (61.8%)
	Stage	II	5 (6.6%)
		III	12 (15.8%)
		IV	59 (77.6%)
	Location	Upper	18 (23.7%)
		Middle	36 (47.4%)
		Lower	22 (28.9%)
	Types of esophageal fistula	EMF	31 (40.8%)
		ETF	42 (55.3%)
		EPF	3 (3.9%)
	Interval between diagnosis and fistula (months)	Mean ± SD	6.55 ± 7.87
		Median (IQR, 25th, 75th)	4 (2,8)
Treatment	Radiation	Y	44 (57.9%)
		N	32 (42.1%)
	Fraction dose	<2 Gy	19 (25.0%)
		≥2 Gy	25 (32.9%)
		Non	32 (42.1%)
	Total dose	<60 Gy	29 (38.2%)
		≥60 Gy	15 (19.7%)
		Non	32 (42.1%)
	Concurrent chemoradiotherapy	Y	21 (27.6%)
		N	23 (30.3%)
		Non	32 (42.1%)
	Chemotherapy	Y	63 (82.9%)
		N	13 (17.1%)
	First‐line chemotherapy regimen	Paclitaxel	50 (65.8%)
		Fluorouracil	13 (17.1%)
		None	13 (17.1%)
	Treatment after fistula	Tube/fistulization	36 (47.4%)
		Stent	36 (47.4%)
		others	4 (5.3%)
Performance status and nutrition	KPS	90	27 (35.5%)
		80	42 (55.3%)
		≤70	7 (9.2%)
	Albumin (g/l)	Mean ± SD	35.05 ± 6.82
		Median (IQR, 25th,75th)	34.30 (29.88,39.92)
	BMI	Mean ± SD	20.36 ± 2.90
		Median (IQR, 25th, 75th)	19.95 (18.60, 21.93)
	Prealbumin (g/l)	Mean ± SD	0.12 ± 0.07
		Median (IQR, 25th, 75th)	0.10 (0.07, 0.162)
Infection and immunity	Peripheral white blood cell count (×10^9^/l)	Mean ± SD	8.39 ± 4.40
		Median (IQR, 25th, 75th)	7.52 (5.37, 10.38)
	Peripheral blood neutrophils (×10^9^/l)	Mean ± SD	6.61 ± 3.87
		Median (IQR, 25th, 75th)	5.80 (3.97, 8.32)
	Peripheral blood lymphocyte (×10^9^/l)	Mean ± SD	1.07 ± 0.64
		Median (IQR, 25th, 75th)	0.99 (0.56, 1.22)
	Neutrophils proportion	>75%	51 (67.1%)
		≤75%	25 (32.9%)
Status		Death	57 (75.0%)
		Live	19 (25.0%)

Abbreviations: BMI, body mass index; EMF, esophageal mediastinal fistula; EPF, esophagopulmonary fistula; ETF, esophagotracheal fistula; IQR, interquartile range; KPS, Karnofsky performance score; SD, standard deviation.

Five patients had not received any treatment before fistula and the other 71 patients were treated with radiotherapy and/or chemotherapy. After a diagnosis of esophageal fistula, 36 patients were treated with stents, 32 with nutrient tubes, four with jejunostomy or gastrostomy, two with both stents and nutrient tubes, one with surgery, and one without any treatment. Anticancer treatment was prescribed in 37 patients, among which 33 patients were treated with chemotherapy with either a single agent or a combination of two agents, four patients by arotinib (a multitarget tyrosine kinase inhibitors) or checkpoint inhibitor. The first choice chemotherapy regimen was paclitaxel (20 cases), followed by capecitabine or 5‐fluorouracil (eight cases) and irinotecan (five cases).

**FIGURE 1:**
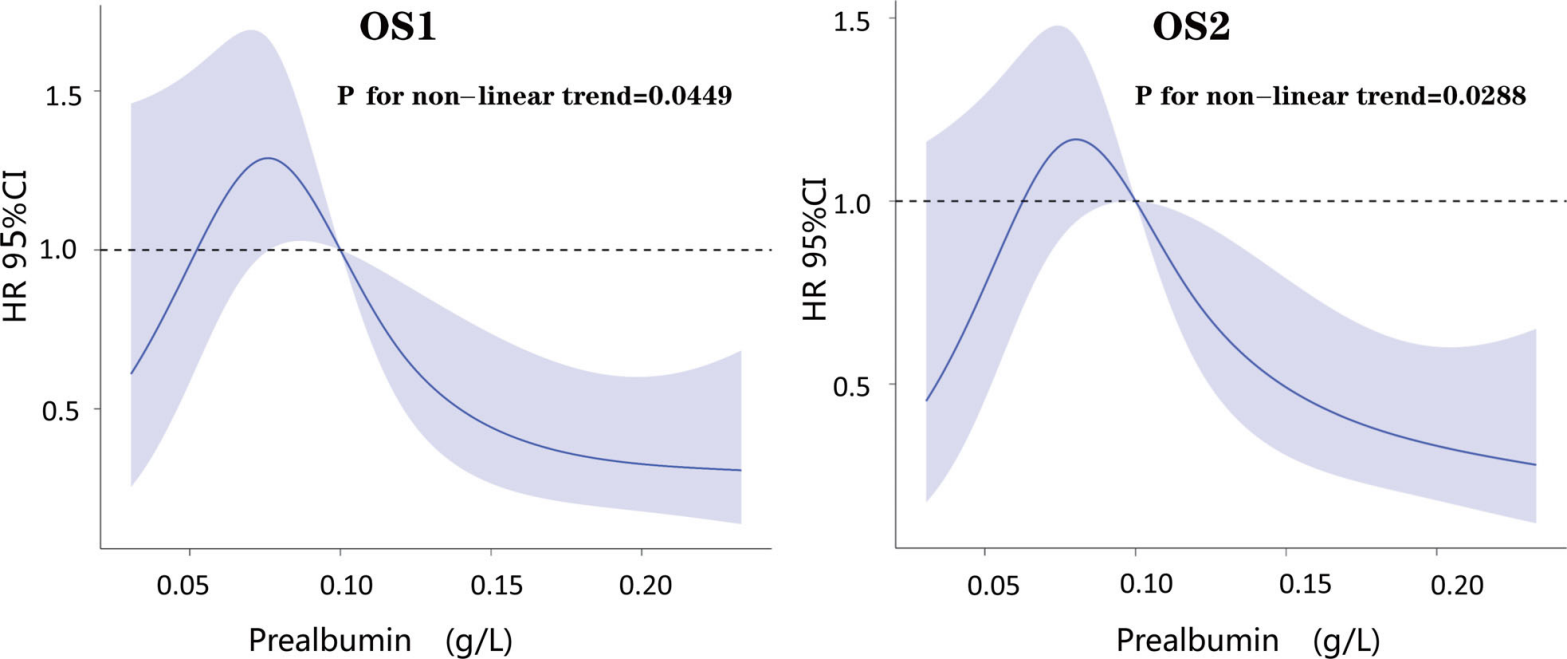
The relationship between serum prealbumin and the risk of death in patients with MEF. Graphs show the hazard ratio (HR; solid lines) and 95% confidence interval (CI, blue areas) describing the association of serum prealbumin with the risk of mortality. Cox regression analysis with a restricted cubic spline approach was conducted to allow nonlinear assessment of the prealbumin in overall survival prediction (a) and post fistula survival prediction (b)

Restrictive cubic spline showed that only prealbumin among numerical variables was nonlinear in the Cox prognostic model of OS1 (*p* = 0.0449) and OS2 (*p* = 0.0288) (Figure 1).

### Survival analysis based on clinical data

A total of 26 suspected prognostic factors in four categories (Tumor, Treatment, Performance Status and nutrition, Infection and immunity) were analyzed by Cox univariate regression model for OS1 and OS2, respectively.

Univariate analysis for OS1 showed significant statistical differences (*p* < 0.05) in KPS, prealbumin, interval between diagnosis of EC and fistula. Multivariate Cox regression analysis incorporated the above variables and age (*p* = 0.126), serum albumin (*p* = 0.0951) and BMI (*p* = 0.138), which indicated age, prealbumin, KPS, interval between diagnosis and fistula were independent prognostic factors (Table 2).

**TABLE 2:** Univariate and multivariate analysis of clinical prognostic factors associated with esophageal fistula

Characteristics	OS1^a^				OS2^b^			
	Univariate analysis		Multivariate analysis		Univariate analysis		Multivariate analysis	
	HR (95% CI)	*p*	HR (95% CI)	*p*	HR (95% CI)	*p*	HR (95% CI)	*p*
Age	1.02 (0.99, 1.05)	0.13	1.05 (1.01, 1.09)	0.02	1.05 (1.02, 1.08)	0.00	1.05(1.01, 1.10)	0.01
Length	0.98 (0.87, 1.10)	0.70			0.97 (0.87, 1.09)	0.63		
Gender								
Female	Reference				Reference			
Male	0.58 (0.26, 1.29)	0.18			0.69 (0.31, 1.52)	0.35		
Stage_T								
T2/T3	Reference				Reference		Reference	
T4	1.22 (0.72, 2.09)	0.46			0.65 (0.39, 1.10)	0.11	0.64 (0.37, 1.11)	0.11
Stage_N								
N0/N1	Reference				Reference			
N2/N3	1.01 (0.60, 1.73)	0.96			0.92 (0.54, 1.56)	0.75		
Stage_M								
M1	Reference				Reference			
M0	1.26 (0.73, 2.17)	0.41			1.24 (0.72, 2.12)	0.44		
Location								
Upper	Reference				Reference			
Middle	0.91 (0.48, 1.74)	0.77			0.98 (0.51, 1.87)	0.94		
Lower	0.99 (0.49, 2.00)	0.97			1.28 (0.64, 2.54)	0.49		
Radiation								
N	Reference				Reference			
Y	0.78 (0.45, 1.36)	0.38			1.37 (0.80, 2.35)	0.26		
Fraction dose								
<2GY	Reference				Reference			
≥2GY	1.03 (0.52, 2.03)	0.94			1.20 (0.62, 2.35)	0.59		
n	1.30 (0.66, 2.58)	0.45			0.81 (0.42, 1.58)	0.54		
Concurrent								
N	Reference				Reference			
Y	1.25 (0.63, 2.48)	0.52			0.82 (0.43, 1.60)	0.57		
n	1.43 (0.74, 2.75)	0.29			0.67 (0.36, 1.24)	0.20		
KPS								
90	Reference		Reference		Reference		Reference	
80	1.55 (0.85, 2.85)	0.16	2.48 (1.23, 5.03)	0.01	2.37 (1.29, 4.35)	0.00	1.94 (0.99, 3.79)	0.05
≤70	3.10 (1.17, 8.18)	0.02	2.36 (0.77, 7.26)	0.13	4.44 (1.65, 11.96)	0.00	4.64 (1.48, 14.53)	0.01
Regimen								
T	Reference				Reference			
F	0.95 (0.47, 1.93)	0.89			1.17 (0.60, 2.30)	0.65		
n	1.67 (0.77, 3.61)	0.19			1.22 (0.56, 2.64)	0.62		
Types								
EMF	Reference				Reference			
ERF	1.09 (0.64, 1.87)	0.743			1.03 (0.61, 1.74)	0.92		
Treatment								
Tube/fistulization	Reference				Reference			
Stent	1.16 (0.68, 2.00)	0.58			1.15 (0.67, 1.97)	0.61		
Others	1.11 (0.33, 3.712)	0.86			1.17 (0.35, 3.90)	0.79		
Albumin	0.96 (0.92, 1.01)	0.10	1.00 (0.94, 1.07)	0.99	0.96 (0.93, 1.00)	0.07	1.07 (1.00, 1.13)	0.04
BMI	0.93 (0.84, 1.02)	0.14	0.91 (0.83, 1.01)	0.09	0.93 (0.84, 1.02)	0.14	0.92 (0.83, 1.01)	0.08
Prealbumin								
0.01–0.1	Reference		Reference		Reference		Reference	
0.11–0.16	0.66 (0.36, 1.21)	0.18	0.80 (0.38, 1.66)	0.54	0.91 (0.50, 1.66)	0.77	1.63 (0.79, 3.39)	0.19
0.16–0.36	0.36 (0.17, 0.77)	0.01	0.33 (0.14, 0.80)	0.01	0.42 (0.21, 0.87)	0.02	0.36 (0.14, 0.89)	0.03
WBC	1.02 (0.96, 1.08)	0.50			1.00 (0.95, 1.06)	0.88		
Leu	1.13 (0.73, 1.75)	0.58			0.73 (0.47, 1.14)	0.17		
Neu	1.02 (0.95, 1.09)	0.59			1.01 (0.95, 1.07)	0.72		
Neu%								
≤75%	Reference				Reference		Reference	
>75%	1.34 (0.74, 2.44)	0.33			2.01 (1.10, 3.68)	0.02	2.58 (1.29, 5.15)	0.01
Interval^c^	0.94 (0.90, 0.98)	0.00	0.88 (0.83, 0.94)	0.00	1.01 (0.99, 1.04)	0.36		

Abbreviations: CI, confidence interval; EMF, esophageal mediastinal fistula; ERF, esophageal respiratory fistula; F, 5‐fluorouracil; HR, hazard ratio; T, taxols.

^a^ Overall survival, the period from the date of admission to the death date.

^b^ Post fistula survival, the period from diagnosis of fistula to death.

^c^ Interval between diagnosis of esophageal cancer and fistula.

Univariate analysis for OS2 showed significant statistical differences (*p* < 0.05) in prealbumin, age, neutrophil proportion, and KPS. Multivariate analysis incorporated the above variables and T stage (*p* = 0.106), albumin (*p* = 0.069), BMI (*p* = 0.136), which indicated age, prealbumin, KPS, albumin and neutrophil proportion were independent prognostic factors (Table 2).

Note: (1) Only 7/76 patients had data on tissue differentiation, and this variable was not included in the analysis. (2) Selection bias may exist for antitumor treatment after esophageal fistula, which was not included in the analysis. (3) Esophageal fistula occurred in eight patients during radiation 2–40 Gy, and radiotherapy was terminated. In order to avoid selection bias, the total radiation dose was not included in the analysis. (4) We used the variance inflation factor (VIF) and Pearson's correlation test to exclude multicollinearity in clinical features, as shown in the Appendix.

### Performance of clinical nomogram

Based on multivariate Cox regression analysis, the clinical prognostic nomogram model was established with independent prognostic factors as parameters.

**FIGURE 2:**
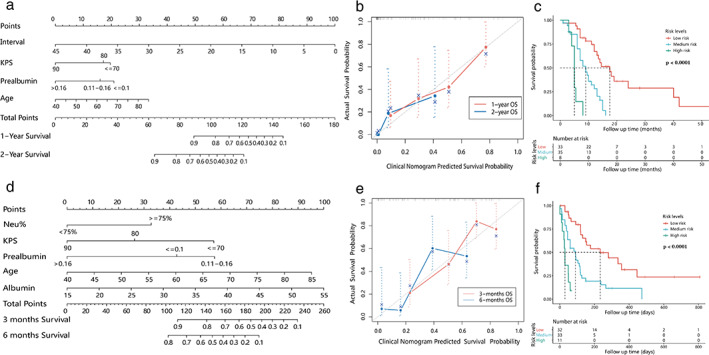
The nomogram of overall survival (a) and post fistula survival (d) developed by clinical prognostic factors. Calibration curves for nomograms (b) overall survival, (e) post fistula survival. The nomogram‐based risk scores calculated by stepwise regression algorithm divided patients into three prognostic groups. Survival analysis of three groups showed significant differences (log‐rank test *p* < 0.0001) (c: overall survival, f: post fistula survival), which showed discrimination of the nomograms

The C‐index of the clinical nomogram for OS1 was 0.719 (95% CI: 0.645–0.793), which was 0.688 with the bootstrap algorithm (1000 iterations). The nomogram and caliberation curve are shown in Figure 2a,b. Risk scores calculated by stepwise regression algorithm ranged from 0.002 to 6.614, and the cutoff value identified by X‐tile was 0.971 and 4.056. All patients were divided into three prognostic groups: high risk (4.056–6.614), medium risk (0.972–4.055), low risk (0.002–0.971). Survival analysis of three groups showed significant differences (*p* < 0.0001) (Figure 2c).

The C‐index of the clinical nomogram for OS2 was 0.722 (95% CI: 0.653–0.791), which was 0.686 with the bootstrap algorithm (1000 iterations). Risk scores were 0.129–6.094, with cutoff value 0.771 and 2.598. The high risk (2.598–6.094), medium risk (0.772–2.597), and low risk (0.129–0.771) groups showed significant survival differences (*p* < 0.0001). The nomogram, caliberation curve and survival plot are shown in Figure 2d–f, respectively.

### Radiomic features selection and radiomic signature (Radscore) construction

A total of 851 radiomic features were extracted from every CT image comprising 52 sets of prefistula and 76 sets of post fistula. Details of feature extraction are shown in the Appendix. A Lasso‐Cox regression model was established for OS1 and OS2, respectively (Appendix Figure S2). The C‐index and number of features with a nonzero coefficient are shown in Table 3. The performance of models of prefistula CT was better than that of post fistula models. Therefore, radiomic signature was based on features selected by Lasso‐Cox regression models for prefistula. The details of selected features and their coefficients are described in Appendix Table S3.

**TABLE 3:** Comparison of the discrimination performance of Lasso‐Cox regression models

Lasso‐Cox regression models		Cases	Number of features	C‐index (95% CI)
Post fistula CT^a^	OS1	76	3	0.705 (0.637–0.773)
	OS2	76	2	0.646 (0.571–0.721)
Prefistula CT^b^	OS1	52	8	0.765 (0.682–0.848)
	OS2	52	4	0.696 (0.609–0.782)

^a^ Contrast‐enhanced CT images performed one month after fistula.

^b^ Contrast‐enhanced CT images performed at initial diagnosis.

**FIGURE 3:**
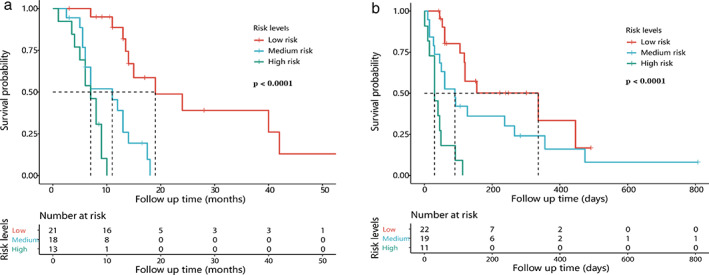
Kaplan–Meier survival curves of prognostic groups divided by radiomics signature (radscore) in overall survival analyses (a) and post fistula analyses(b). Significant differences were observed in both (log‐rank test *p* < 0.0001), which indicated that radiomic signature was significantly associated with survival of MEF

Radscores of OS1 ranged from 0.103 to 1.412, with cutoff values of 0.300 and 0.550, dividing patients into three prognosis groups including high risk (0.550–1.412), medium risk (0.301–0.549), and low risk (0.103–0.300). Survival analysis of the three groups showed significant differences (*p* < 0.0001) (Figure 3a).

Radscores of OS2 ranged from 0.015 to 0.414, with cutoff values of 0.125, and 0.198, dividing patients into high risk (0.198–0.414), medium risk (0.126–0.197), low risk (0.015–0.125), which showed significant survival differences (*p* < 0.0001) (Figure 3b).

### Performance of joint nomogram

**FIGURE 4:**
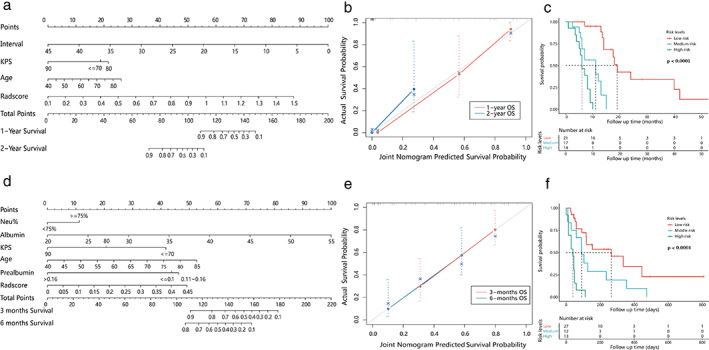
Clinical radiomic nomogram for overall survival (a) and post fistula survival (d). Calibration curves of the joint nomograms (b) overall survival, (c) post fistula survival). The nomogram‐based risk scores divided patients into three prognostic groups. Survival analysis showed significant differences (log‐rank test *p* < 0.0001) (c: overall survival, f: post fistula survival), which showed excellent discrimination of the nomograms

Joint nomograms combining radscores and independent clinical prognostic factors, were established for OS1 and OS2, respectively (Figure 4a,d).

The C‐index of OS1 and OS2 was 0.831 (0.757, 0.905) and 0.77 (0.686, 0.854), respectively which was 0.803 and 0.717 with the bootstrap algorithm (1 000 iterations). The calibration curve showed that the joint nomograms outperformed the clinical ones (Figure 4b,e).

Risk scores of OS1 calculated by stepwise regression algorithm was 0.001–14.563 with cutoff value 0.585 and 3.248. The high risk (3.248–14.563), medium risk (0.586–3.247), and low risk (0.001–0.585) groups showed significant survival differences (*p* < 0.0001) (Figure 4c).

Risk scores for OS2 were 0.203–9.549, with cutoff values of 0.847 and 1.858. Significant survival differences were found in the high (1.858–9.549), medium (0.848, 1.857), and low risk (0.203–0.847) groups (*p* < 0.0001) (Figure 4f).

## DISCUSSION

In the present study, we analyzed the clinical prognostic factors for esophageal fistula and developed clinical‐radiomic nomograms for survival of MEF. To the best of our knowledge, this is the first nomogram incorporating radiomics and clinical factors for the prognosis of MEF.

The median OS of patients with MEF in this group was 11 months, and median survival time after fistula was 113 days, which was similar to that reported in previous studies^7^ and significantly lower than that of nonfistula patients at the same stage.^12^ A total of 26 clinical factors on tumor, treatment, nutrition and infection were analyzed. Univariate and multivariate analysis showed that stage, radiotherapy and chemotherapy had no significant effect on the prognosis of patients with malignant esophageal fistula, whereas nutrition, age, KPS and infection played a more important role.

Nutritional status is an important predictor and prognostic factor for esophageal fistula. Watanabe et al. revealed that a BMI below 20 kg/m^2^ is a risk factor for esophageal fistula formation.^13^ However, its role in prognosis has not been confirmed. In the present study, both serum albumin and prealbumin were independent prognostic factors of post fistula survival, and furthermore prealbumin was an independent prognostic factor of overall survival. Serum albumin outweighed the other independent factors in the clinical nomogram for post fistula survival. The results above illustrate that nutrition plays an important part in the prognosis of MEF. Interestingly, prealbumin correlated with survival in a nonlinear manner. A nonlinear correlation between prealbumin and survival was also found in a study on hepatocellular carcinoma,^14^ but the reason for this cannot be explained at present.

Age was an independent prognostic factor for both overall survival and post fistula survival, which may be explained by poor tolerance and more complications in elderly patients. A study on postoperative survival of esophageal cancer found that the short‐ and long‐term mortality increased with age, but was not affected by other prognostic factors.^15^

Neutrophil proportion was another independent prognostic factor for post fistula survival. Esophageal fistula leads to a greater risk of infection. Leakage of digestive fluid and food into the mediastinum or respiratory tract can cause an uncontrollable abscess and a systemic inflammatory response. It had been reported that the mortality of patients from a mediastinal abscess is as high as 40%.^16^ In our study, the risk of death was 2.58 times higher in patients with a neutrophil proportion >75% within one week after the diagnosis of a fistula. Therefore, in the early phase of esophageal fistula, a significant increase in the neutrophil proportion is suggestive of the existence of infection and a poor prognosis.

The purpose of treatment for MEF is to restore food intake and prevent flow of digestive juices through the fistula. The measures include surgical resection/repair of fistula, gastrostomy/jejunostomy, nutrition tube implantation, stent implantation, and best supportive care. There are few patients who are candidates for esophagectomy. Most patients are at an advanced stage of cancer with nutritional depletion and pulmonary sepsis being common complications at presentation.^17^ Even if patients can be treated surgically, the mortality rate is high. In a previous study, it was reported that the complication rate was 40% and postoperative mortality was 14.3%.^18^ It is generally not recommended to perform such procedures in a palliative situation if a patient has a limited lifespan. For some strictly selected patients, surgery may be an optimal choice, but at present there is a lack of high‐level evidence to support this. A feeding tube has been reported to be the preferred treatment before a stent and is most widely used because it can significantly reduce the incidence of aspiration pneumonia or mediastinitis, and establish a way of supplying nutrition.^19^ However, the quality of life for patients being fed via nasogastric tube is poor, and this method cannot completely avoid digestive fluid from the fistula entering the chest or respiratory tract. Stents can completely close the fistula allowing patients to eat through the mouth, but they might cause bleeding and necrosis due to compression of surrounding tissue. Moreover, complications of retrosternal pain and chest discomfort after stent placement have been reported in almost 50% of patients.^20^ Studies had found that stents can benefit patients by reliving symptoms of cough and suffocation, but whether it can improve survival has not yet been confirmed.^2^, ^20^ In this study, there was no significant difference in survival time between patients treated with stents, or a nutrition tube. Antitumor treatment after a diagnosis of MEF has always been controversial. It is generally believed that malignant esophageal fistula cannot be healed, and antitumor treatment is a cause of its occurrence, and therefore symptomatic support treatment is preferable in most cases. However, some patients have also been reported to achieve long‐term survival after treatment with radiotherapy and chemotherapy, but this is only applicable to patients with a good performance status.^15^ In our study, 33 patients were treated with single or combined chemotherapy after diagnosis of an esophageal fistula. Because these patients had a better KPS and a more positive treatment attitude, we did not conduct survival analysis on this variable in order to avoid selection bias.

The performance of a clinical nomogram with a C‐index of 0.719 for OS1 and 0.722 for OS2 is barely satisfactory. In order to improve performance, multifeature‐based radiomic signatures and joint prediction nomograms incorporating radiomic signature and clinical factors have been developed. The radiomic signature is an independent prognosis factor for OS1 and OS2, which stratifies patients into risk groups with significant differences in survival. The nomogram combining radiomic signature with clinical factors performed better than the clinical model, which revealed incremental value of radiomics for individualized survival prediction in patients with MEF. According to the hypothesis of radiomics, the difference with radiomic features is the macroscopic manifestation of gene differential expression which leads to heterogeneity in tumor presentation and prognosis. Foley et al. found that the texture features of PET images were associated with outcomes of EC.^21^ Another study revealed CT imaging features could be used to stratify patients with esophageal cancer, and had a correlation with tumor metabolism, stage and survival.^22^ Compared with clinical predictors, the radiomic signature (radscore) was dominant in the joint nomogram (as shown in Figure 4a,d). A possible interpretation was that the high dimensional data mined from tumor images can better reflect the heterogeneity of tumors. Improvement of accuracy in models combining radiomics with clinical factors has previously been reported in many studies.^23^, ^24^ For a more parsimonious prediction model, a risk score was calculated by a stepwise regression algorithm. The risk score based on the joint nomogram divided patients into three groups with a significant difference in prognosis, which further confirmed the excellent discrimination of the joint nomogram.

To obtain the optimal radiomic signature, a Lasso‐Cox algorithm was used to screen features from contrast CT performed before and after a fistula, respectively. The Lasso‐Cox regression model of prefistula CT features had a better discrimination than that of post fistula. A possible reason for this would be that CT images before treatment can better reflect the heterogeneity of the tumor since they are not affected by the treatment and local inflammatory response.

There are a few limitations. First, with the retrospective study design, there were many confounding factors affecting outcomes, and the results need to be further confirmed by prospective studies. Second, because of the relatively small sample size, we did not set up an external validation group, but used the bootstrap method (1000 iterations) within the primary group for validation. Finally, in order to avoid selection bias, tumor differentiation, total dose of radiotherapy and antitumor treatment after fistula were not included in the analysis. Further cohort studies are needed to identify these potential influencing factors.

In conclusion, the study presents nomograms incorporating independent clinical risk factors and radiomic signature to predict the prognosis of malignant esophageal fistula. This risk classification system has the potential to guide therapeutic decisions for patients with MEF.

## CONFLICT OF INTEREST

All authors declare no conflict interest.

## Supporting information


**Appendix S1**: Supporting information
