# Supplementary material for: Development and validation of a prognostic nomogram for malignant esophageal fistula based on radiomics and clinical factors
Source: Thorac Cancer. 2021 Oct 14;12(23):3110–20. doi: 10.1111/1759-7714.14115 (PMC8636210; doi:10.1111/1759-7714.14115)
Supplement: Supplementary file 1 — Appendix S1: Supporting information [file TCA-12-3110-s001.docx]

Development and validation of a prognostic nomogram for malignant esophageal fistula based on radiomics and clinical factors

Chao Zhu et al.

**Fig.S1 Flowchart of the study**


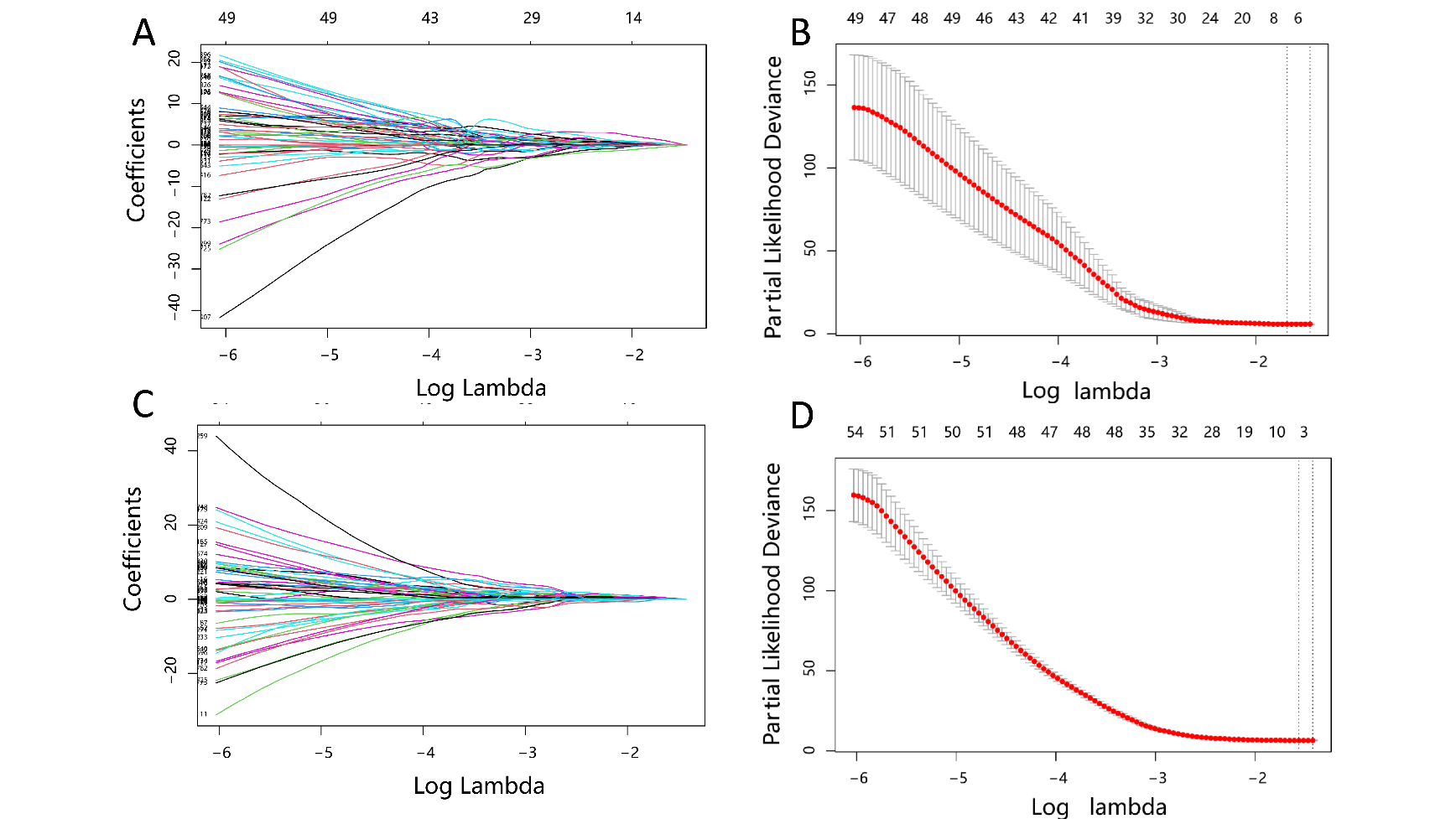


**Fig.S2**  Feature selection using the least absolute shrinkage and selection operator (LASSO) with a Cox regression model. In the model, the coefficient of variables tends to zero with the increasing of parameter Lambda **(Overall survival:A,Post-fistula survival:C).**Tuning Lambda based on minimum criteria using 10-fold cross-validation, the partial likelihood deviance regard to Lambda was plotted **(Overall survival:A,Post-fistula survival:C)**. The left vertical dotted line represents the one with the minimum deviance among all the lambda values, the right vertical line represents the Lambda of the simplest model within a 1-se of minimum value. In this study, minimum-deviance Lambda was chosen, and 8 radiomics features for OS1**(B)**,4 features for OS2**(D)** were filtered.

**Tab.S1 Packages in R used in this study**

| **Methods** | **Package** | **Version** |
| --- | --- | --- |
| LASSO | glmnet | 4.0-1 |
| Cox proportional hazards regression analyse | survival | 3.2-7 |
| Kaplan-Meier method, Logrank test | survminer | 0.4.8 |
| Nomogram, calibration curve, Restricted cubic spline | rms | 6.0-1 |
| Statistical chart drawing | ggplot2 | 3.3.2 |

**Radiomic Features Extraction**

ALL extracted radiomics features can be divided into 4 categories. The ‘shape’ category including 14 features, which represents the shape and size of region of interest (ROIs) in 2D and 3D spaces. The ‘first-order’ represents statistical eigenvalues of voxel intensity, including mean, maximum, and minimum values. Textural features including Glcm(GrayLevelCooccurenceMatrix), Glrlm(GrayLevelRunLengthMatrix), Glszm(GrayLevelSizeZoneMatrix), Gldm(GrayLevelDependenceMatrix), Ngtdm(NeighbouringGrayToneDifferenceMatrix) were calculated from several statistical matrices and described the arrangement of voxel intensity within ROIs. Wavelet-based features were defined as first-order and texture features calculated from eight wavelet decompositions of the original CT image. Therefore, the total number of radiomic features could be calculated as 14+(18+24+16+16+14+5) +(18+24+16+16+14+5)×8=851.

**Tab.S2. Features used in this study.**

| **Categories** | **N** | **Features** |
| --- | --- | --- |
| Shape | 14 | Elongation  Flatness  LeastAxisLength  MajorAxisLength  Maximum2DDiameterColumn  Maximum2DDiameterRow  Maximum2DDiameterSlice  Maximum3DDiameter  MeshVolume  MinorAxisLength  Sphericity  SurfaceArea  SurfaceVolumeRatio  VoxelVolume |
| firstorder | 18 | 10Percentile  90Percentile  Energy  Entropy  InterquartileRange  Kurtosis  Maximum  MeanAbsoluteDeviation  Mean  Median  Minimum  Range  RobustMeanAbsoluteDeviation  RootMeanSquared  Skewness  TotalEnergy  Uniformity  Variance |
| Glcm(GrayLevelCooccurenceMatrix) | 24 | Autocorrelation  ClusterProminence  ClusterShade  ClusterTendency  Contrast  Correlation  DifferenceAverage  DifferenceEntropy  DifferenceVariance  Id  Idm  Idmn  Idn  Imc1  Imc2  InverseVariance  JointAverage  JointEnergy  JointEntropy  MCC  MaximumProbability  SumAverage  SumEntropy  SumSquares |
| Glrlm(GrayLevelRunLengthMatrix) | 16 | GrayLevelNonUniformity  GrayLevelNonUniformityNormalized  GrayLevelVariance  HighGrayLevelRunEmphasis  LongRunEmphasis  LongRunHighGrayLevelEmphasis  LongRunLowGrayLevelEmphasis  LowGrayLevelRunEmphasis  RunEntropy  RunLengthNonUniformity  RunLengthNonUniformityNormalized  RunPercentage  RunVariance  ShortRunEmphasis  ShortRunHighGrayLevelEmphasis  ShortRunLowGrayLevelEmphasis |
| Glszm(GrayLevelSizeZoneMatrix) | 16 | GrayLevelNonUniformity  GrayLevelNonUniformityNormalized  GrayLevelVariance  HighGrayLevelZoneEmphasis  LargeAreaEmphasis  LargeAreaHighGrayLevelEmphasis  LargeAreaLowGrayLevelEmphasis  LowGrayLevelZoneEmphasis  SizeZoneNonUniformity  SizeZoneNonUniformityNormalized  SmallAreaEmphasis  SmallAreaHighGrayLevelEmphasis  SmallAreaLowGrayLevelEmphasis  ZoneEntropy  ZonePercentage  ZoneVariance |
| Gldm(GrayLevelDependenceMatrix) | 14 | DependenceEntropy  DependenceNonUniformity  DependenceNonUniformityNormalized  DependenceVariance  GrayLevelNonUniformity  GrayLevelVariance  HighGrayLevelEmphasis  LargeDependenceEmphasis  LargeDependenceHighGrayLevelEmphasis  LargeDependenceLowGrayLevelEmphasis  LowGrayLevelEmphasis  SmallDependenceEmphasis  SmallDependenceHighGrayLevelEmphasis  SmallDependenceLowGrayLevelEmphasis |
| Ngtdm(NeighbouringGrayToneDifferenceMatrix) | 5 | Busyness  Coarseness  Complexity  Contrast  Strength |

**Tab.S3 Radiomics features filtered by LASSO-Cox regression analyse**

| **Selected radiomics features associated with overall survival (OS1)** | **Coefficient** |
| --- | --- |
| wavelet.LHH_firstorder_Kurtosis | -0.026 |
| wavelet.HLL_glszm_GrayLevelVariance | 0.325 |
| wavelet.HLH_glszm_LargeAreaLowGrayLevelEmphasis | 0.027 |
| wavelet.HLH_ngtdm_Contrast | 1.138 |
| wavelet.HHH_glszm_GrayLevelNonUniformityNormalized | -0.495 |
| wavelet.LLL_firstorder_90Percentile | 0.029 |
| wavelet.LLL_firstorder_RootMeanSquared | 0.401 |
| wavelet.LLL_glszm_GrayLevelVariance | 0.406 |
| **Selected radiomics features associated with post-fistula survival (OS2)** | **coefficient** |
| original_glcm_Imc1 | -0.001 |
| wavelet.HLL_firstorder_Median | 0.0933 |
| wavelet.HLH_ngtdm_Busyness | 0.209 |
| wavelet.LLL_glszm_GrayLevelVariance | 0.307 |

**Multicollinearity test for clinical features**

The serious multicollinearity in regression analysis (closely associated with each other) affects the stability of the analytical model. However, moderate multicollinearity does not pose a problem. Two statistical methods are usually used to judge serious multicollinearity: variance expansion factor (VIF > 10) and correlation coefficient (R^2^ > 0.8 / 0.9).

Among these variables, only WBC was highly correlated with Neu (R^2^=0.98), and the correlation coefficients of other variables were between 0.61 and 0.12, which did not constitute serious multicollinearity(Fig S3). WBC and Neu did not appear in a multivariate regression analysis at the same time, so the results were reliable.

In order to further exclude multicollinearity, we use a variance inflation factor (VIF) for verification. The VIF in the Cox multivariate regression model of overall survival(OS1) was between 1.34 and 1.42, and that was 6.72 in post-fistula survival analysis(OS2).

References for Multicollinearity:

Jong Hae Kim. Multicollinearity and misleading statistical results. Korean J Anesthesiol,2019 Dec;72(6):558-569.


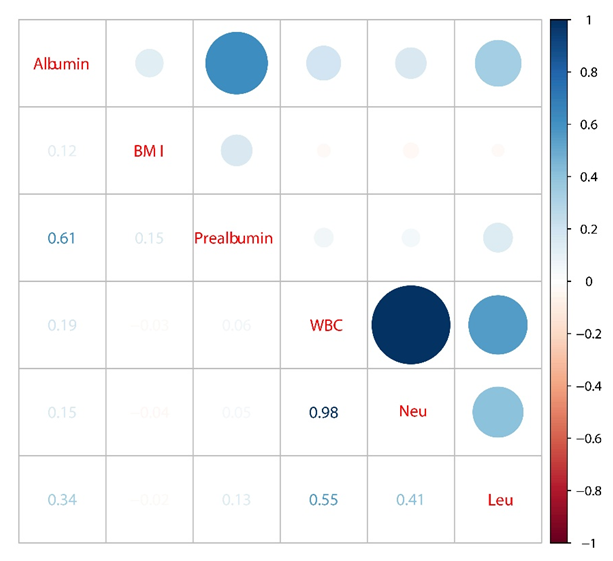


**Fig.S3 Correlation between clinical features. The size and color depth of the dot indicate the correlation coefficients**
